# 

**Published:** 1861-09

**Authors:** 


### CHICAGO MEDICAL JOURNAL.

**Terms, $2.00 per Annum.**

### CONTENTS OF THE SEPTEMBER NUMBER.

SELECTED.

PAGE.

An Inquiry into the Therapeutic Value of Sulphate of Magnesia, Oil of Turpentine and Calomel, in Inflammation of Intestinal Mucous Membrane, especially in Dysentery. By Wm. Henry Thayer, M. D., 501

Sanitary Science in the Camp—Being the Conclusions of the Commission appointed by the British Government to Inquire into the Sanitary Condition of the Army in the Crimea. By George W. Wilde, M. D., of London.......................................... 510

Description of the "Brigade Case," designed by H. S. Hewit, M. D., formerly Surgeon U. S. A.......................................... 517

The Chemical Treatment of Diseases. By. C. B. Hall, M. D............. 519

Intercourse as Affecting Uterine Diseases, and their Treatment. By Augustus K. Gardner, M. D., Professor of Clinical Midwifery and Diseases of Women.......................................... 524

Alcoholic Drinks and Phthisis.......................................... 557

ORIGINAL COMMUNICATIONS.

Epilepsy Caused by the Ascaris Lumbricoides. By M. M. Eaton, M. D., of Peoria.......................................... 530

A Couch for a Patient with a Fractured Femur. My Elmer Nichols, Medical Student.......................................... 533

EDITORIAL.

Editorial and Miscellaneous.......................................... 539

